# Water Sources and Biomass Allocation Characteristics of Dominant Species in the Desert Riparian Forest Along the Keriya River, Northwest of China

**DOI:** 10.1002/ece3.73686

**Published:** 2026-05-20

**Authors:** Yue Dai, Zhuanxiong Ye, Anwar Abdureyim, Mengmeng Tang, Kerui Yao

**Affiliations:** ^1^ College of Geography and Remote Sensing Science Xinjiang University Urumqi China; ^2^ Xinjiang Field Scientific Observation and Research Station for the Oasisization Process in the Hinterland of the Taklamakan Desert Yutian China; ^3^ Xinjiang Key Laboratory of Oasis Ecology Xinjiang University Urumqi China; ^4^ College of Ecology and Environment Xinjiang University Urumqi China

**Keywords:** biomass allocation, riparian forest, root distribution, stable isotopes, water source

## Abstract

*Populus euphratica* and *Tamarix ramosissima* are dominant species in desert riparian forests, playing a pivotal role in sustaining biodiversity and ecosystem functionality. However, the nature and dynamics of their interspecific interactions across heterogeneous habitats remain insufficiently understood. This study examined the adaptive strategies of co‐occurring *P. euphratica* and *T. ramosissima* seedlings along the Keriya River in the Taklamakan Desert, focusing on water source utilization, water use efficiency (WUE), root distribution, and biomass allocation. Both species relied predominantly on shallow soil water (0–40 cm depth). *P. euphratica* displayed a significantly higher leaf *δ* ^13^C value (−29.21‰) than *T. ramosissima* (−30.51‰), reflecting greater WUE. Root biomass was heavily concentrated in the 0–40 cm layer, accounting for 79.83% of total root biomass in *P. euphratica* and 71.52% in *T. ramosissima* . Analysis of the root: shoot ratio in relation to total biomass revealed contrasting ontogenetic allocation strategies: *P. euphratica* exhibited no significant trend, indicating stable biomass partitioning between roots and shoots throughout development, whereas *T. ramosissima* displayed a significant positive correlation, reflecting a progressive increase in belowground biomass investment with increasing plant size. These divergent resourceacquisition strategies suggest that *T. ramosissima* may gain a competitive advantage through rapid root proliferation, particularly under fluctuating hydrological conditions. Collectively, these findings enhance our understanding of functional niche differentiation and community assembly processes in arid‐zone riparian ecosystems and provide ecologically informed guidance for riparian restoration, particularly regarding species selection and adaptive water management.

## Graphical abstract

Our results highlight water sources and biomass allocation characteristics of dominant species in the desert riparian forest along the Keriya River. These findings provide a scientific basis for predicting the developmental dynamics within desert riparian forest communities.

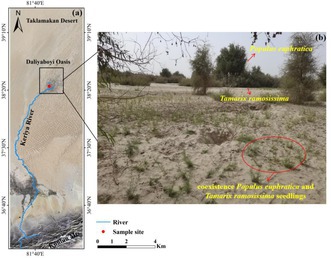

## 1 Introduction

Water availability is the primary factor limiting plant survival and growth in arid and semi‐arid regions, with root distribution playing a critical role in accessing soil moisture (Pokhrel et al. 2021; Zhao et al. 2025; Tariq et al. 2026). Plants in these environments exhibit diverse water‐use strategies shaped by soil water dynamics (Green et al. 2019), root plasticity (Bardgett et al. 2014; Fan et al. 2017), and developmental stage (Pei et al. 2023). Understanding these strategies is essential for predicting plant responses to environmental change. Stable isotope techniques provide a powerful tool for quantifying plant water sources, as minimal fractionation occurs during root water uptake, allowing xylem water isotopic composition to be linked to potential sources (Dawson et al. 2002; Evaristo et al. 2015; Sprenger et al. 2016). Leaf carbon isotope composition (*δ* ^13^C) serves as an integrated proxy for long‐term water use efficiency (WUE), with higher values indicating greater WUE under drought stress (Ehleringer 1993; Cao et al. 2020). In parallel, biomass allocation patterns reflect plant adaptive responses to resource limitation; plants often increase belowground investment under drought or competition to enhance resource acquisition, whereas favorable conditions promote aboveground growth (Poorter et al. 2012; Tumber‐Dávila et al. 2022).

In arid regions of Eurasia and North America, desert riparian forests dominated by *Populus* and *Tamarix* species play a critical role in maintaining ecosystem stability, windbreaks, and sand fixation (Horton et al. 2001; Lite and Stromberg 2005; Preislerová et al. 2024). The ecological dynamics of these two genera have been extensively studied globally (Glenn and Nagler 2005; Akhmedov et al. 2025). In North America, where *Tamarix ramosissima* was introduced in the 1800s and has since become a widespread invader, extensive research has examined its competitive dynamics with native *Populus* species (DiTomaso 1998; Nippert et al. 2010). Hydrological alterations, particularly groundwater decline and flow regulation, strongly favor *Tamarix* over *Populus* by shifting competitive balances (Stromberg et al. 2007; Merritt and Poff 2010). Experimental work has further demonstrated that *Tamarix* seedlings exhibit greater drought tolerance and flexible water‐use strategies under variable water availability, yet are not inherently superior competitors under all conditions; rather, anthropogenic modifications to flood regimes and groundwater levels create conditions that favor *Tamarix* establishment (Sher and Marshall 2003). Additionally, Lavaine et al. (2015) examined the use of European Tamaricaceae in soil bioengineering, highlighting the ecological utility of *Tamarix* in stabilizing dryland riparian zones. This global body of research, spanning from invasion biology to native range ecology and applied restoration, highlights the competitive strength and environmental adaptability of *Tamarix*, providing essential context for understanding its interactions with coexisting *Populus* species.

In the Central Asian context, desert riparian forests, particularly those in the Tarim Basin, are primarily composed of *Populus euphratica* and *T. ramosissima* (Ling et al. 2019). In natural floodplains, their seedlings often co‐occur with high niche overlap during early establishment (Li et al. 2013). While previous research has documented species‐specific differences in adult water relations, such as tighter coupling of *P. euphratica* water status to groundwater levels versus greater reliance of *T. ramosissima* on deep soil moisture (Gries et al. 2003; Li et al. 2019; Wan et al. 2022), few studies have examined the interactive strategies of their seedlings under natural field conditions. Existing seedling studies have largely relied on controlled pot experiments (Li et al. 2013; Wu et al. 2018), limiting our understanding of in situ coexistence mechanisms during this vulnerable life stage, when water stress and competition critically shape recruitment and community assembly.

The Keriya River flows through the Taklamakan Desert hinterland, maintaining a naturally functioning riparian ecosystem at its terminus, the Daliyaboyi Oasis, with minimal anthropogenic disturbance (Wang et al. 2022). This oasis represents one of the few remaining sites in the Tarim Basin where natural hydrological processes (seasonal floods and groundwater fluctuations) remain largely intact, providing an ideal setting to examine plant‐water relations without the confounding effects of artificial water diversion or land‐use change (Shi et al. 2024; Zhang et al. 2024). Moreover, the site supports natural co‐occurrence of *P. euphratica* and *T. ramosissima* seedlings under extreme aridity, with similar microsite conditions across the sampling area, allowing for direct comparisons of species‐specific strategies (Abdureyim et al. 2024). These characteristics make the Daliyaboyi Oasis a unique natural laboratory for investigating interspecific interactions during the critical seedling stage, a research context that cannot be replicated in pot experiments or anthropogenically altered riparian corridors.

In this study, we conducted an in situ investigation of the adaptive strategies of cooccurring *P. euphratica* and *T. ramosissima* seedlings. Given their high niche overlap at the seedling stage (Li et al. 2013; Wu et al. 2018), we hypothesized that (i) the two species share similar water sources under the same hydrological conditions, and (ii) to reduce competition, they diverge in biomass allocation patterns as they grow, thereby facilitating their coexistence. By testing these hypotheses, we aim to elucidate how seedling‐stage interactions contribute to the coexistence of these dominant species and to inform restoration practices in desert riparian ecosystems.

## 2 Materials and Methods

### 2.1 Study Area

The Keriya River originates in the Kunlun Mountains and terminates in the Daliyabuyi Oasis (38.244°–38.627° N, 81.688°–82.161° E), the largest naturally functioning oasis in the hinterland of the Taklamakan Desert (Wang et al. 2022). Covering an area of 324 km^2^, the oasis spans an elevation range of 1100–1400 m and exhibits a warm temperate arid desert climate, characterized by a mean annual temperature of 11.6°C, annual precipitation < 50 mm, and potential evapotranspiration exceeding 2480 mm (Zhang et al. 2024). Vegetation is dominated by *P. euphratica* trees, along with shrubs of *T. ramosissima* and *T. chinensis* , and associated herbaceous species, including *Phragmites australis* , *Karelinia caspia*, and *Sophora alopecuroides*. Soils at the sampling sites are classified as forest‐shrub meadow soils, exhibiting a textural gradient from loam to sand. The study area lies west of the oasis center, where surface water from the Keriya River is predominantly distributed (Zhang et al. 2024); this results in relatively high soil moisture levels, thereby providing favorable conditions for investigating interspecific interactions among seedlings.

To characterize the general population structure of seedlings at the study site, three 1 m × 1 m quadrats were randomly established for assessing seedling density and morphological traits (height, crown width, basal diameter). These descriptive statistics are summarized in Table 1. It is important to note that these quadrats were used solely for population characterization; the primary functional trait data (root distribution, biomass allocation, and stable isotope analysis) were collected from a separate set of 12 well‐established individuals per species, sampled across a larger area to ensure representativeness.

**TABLE 1:** Morphological characteristics of *P. euphratica* and *T. ramosissima* seedlings derived from quadrat surveys in the Daliyabuyi Oasis.

Species	Height (cm)	Crown width (cm)	Basal stem diameter (mm)	Density (m^−2^)
*P. euphratica*	18.76 ± 6.23	14.05 ± 3	2.51 ± 0.21	7 ± 1
*T. ramosissima*	16.28 ± 3.67	10.14 ± 1.5	2.12 ± 0.22	27 ± 6

*Note:* Data are mean ± SE based on measurements from three 1 m × 1 m quadrats, with each quadrat containing multiple individuals per species.

### 2.2 Sample Collection

#### 2.2.1 Plant Sample Collection

**FIGURE 1:**
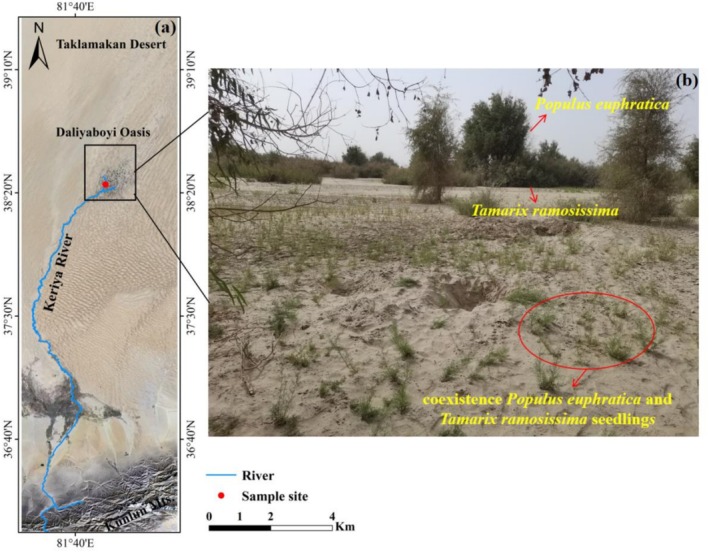
The sampling site located in the Daliyaboyi Oasis at the tail of the Keriya River, situated in the hinterland of the Taklamakan Desert (a), along with images depicting the coexistence of *P. euphratica* and *T. ramosissima* seedlings (b).

Field sampling was conducted in late July 2022 within a naturally cooccurring stand of *P. euphratica* and *T. ramosissima* seedlings located in the Daliyaboyi Oasis (Figure 1). This site was selected to represent minimal anthropogenic disturbance and typical habitat conditions characteristic of the region. No rainfall or surface runoff occurred during the seven days preceding sampling, thereby minimizing confounding hydrological effects.

Twelve well‐established individuals per species, each exhibiting a basal stem diameter of 0–5 mm, were selected for root excavation and subsequent biomass analysis. This sample size (*n* = 12 per species) aligns with established ecological sampling guidelines for plant functional trait studies, which generally recommend sampling 5–10 individuals per species to adequately represent intraspecific variation (Pérez‐Harguindeguy et al. 2016; Wigley et al. 2020). Our design, sampling 12 individuals per species, therefore falls within and slightly exceeds this recommended range. To minimize sampling bias, all selected seedlings were naturally regenerated, ensuring exposure to comparable microsite conditions. Prior to excavation, plant height, crown width, and basal stem diameter were measured using a standard tape measure and digital vernier calipers. Mean morphological measurements (±standard error) were as follows: *P. euphratica*: height 28.42 ± 5.13 cm, crown width 17.86 ± 4.13 cm, basal diameter 3.01 ± 0.38 mm; *T. ramosissima* : height 32.02 ± 5.74 cm, crown width 11.35 ± 1.93 cm, basal diameter 2.95 ± 0.43 mm.

A ring ditch with a diameter of 2–3 m (approximately 5–10 times the seedling crown width) was excavated around each selected plant to provide sufficient working space. Soil was removed in sequential 20‐cm vertical increments, starting from the surface and proceeding downward. At each increment, loose soil was carefully extracted using hand trowels and shovels, working outward from the stem to avoid damaging the root system. As excavation progressed, the primary root was progressively exposed, and lateral roots were carefully traced from their emergence point on the primary root outward. Each lateral root was followed until its terminal branches were fully exposed or until it could be clearly identified as belonging to the target plant. All exposed roots were kept intact throughout the excavation process.

All plant material was oven‐dried at 65°C for 48 h and subsequently weighed to determine aboveground and belowground biomass. The root: shoot ratio (R:S) was calculated as the dry mass of below‐ground biomass divided by the dry mass of above‐ground biomass (Jackson et al. 1996; Poorter et al. 2012). For vertical root distribution analysis, we selected eight individuals per species with a basal stem diameter of 2–5 mm, as preliminary excavations indicated that seedlings with basal diameters < 2 mm lacked roots below 60 cm depth.

For leaf carbon isotope (*δ* ^13^C) analysis, six additional individuals per species (basal diameter 2–5 mm) were sampled. Fully expanded leaves were collected from mid‐canopy positions, oven‐dried at 65°C to constant mass, ground to pass a 100‐mesh sieve, and stored in sealed bags pending isotopic analysis.

All field sampling was conducted with permission from the local forestry authorities and in accordance with institutional guidelines for ecological research.

#### 2.2.2 Soil Sample Collection

Adjacent to each excavated plant, soil cores were collected using a 10‐cm‐diameter hand auger at 20‐cm depth intervals, continuing down to the phreatic zone (water table). Three replicate cores were obtained per depth interval. Each core was subdivided into two subsamples: one was sealed in a 100‐mL polyethylene bottle and stored frozen for stable isotope analysis; the other was placed in a tin box for gravimetric soil water content (SWC) determination by oven drying at 105°C to constant mass.

#### 2.2.3 Isotope Analysis

For xylem water isotope analysis, suberized twigs (0.1–0.3 cm in diameter, 3–5 cm in length) were collected from eight individuals per species (basal stem diameter: 2–5 mm), immediately sealed in glass vials using Parafilm, and stored at −20°C until analysis. Water was extracted via cryogenic vacuum distillation (LI‐2100, LICA, Beijing, China), filtered through 0.22‐μm syringe filters, and analyzed using a liquid water isotope analyzer (LWIA DLT‐100, Los Gatos Research, USA). Isotopic results are reported in *δ*‐notation (‰) relative to Vienna Standard Mean Ocean Water (VSMOW), with analytical precisions of ±0.50‰ for *δ* ^2^H and ±0.10‰ for *δ* ^18^O. Methanol and ethanol contamination was corrected using empirically derived standard curves (Schultz et al. 2011). Leaf *δ* ^13^C values were determined using a stable isotope ratio mass spectrometer (DELTA‐V Advantage, Thermo Fisher Scientific, Germany), with an analytical precision of ±0.30‰, and reported relative to the Pee Dee Belemnite (PDB) standard. Isotope ratios were calculated as follows:

(1) δsample000=RsampleRstandard−1×1000

where *R*_sample_ and *R*_standard_ denote the isotopic ratios (^2^H/^1^H, ^18^O/^16^O or ^13^C/^12^C) of the sample and reference standard, respectively.

The Global Meteoric Water Line (GMWL), defined by Craig (1961) as *δ* ^2^H = 8*δ* ^18^O + 10, represents the long‐term global average relationship between *δ* ^2^H and *δ* ^18^O in precipitation, reflecting equilibrium isotope fractionation during evaporation and condensation under minimal kinetic effects (Dansgaard 1964). The Local Meteoric Water Line (LMWL) for the study region was established using regional precipitation isotope data, yielding the equation *δ* ^2^H = 7.2*δ* ^18^O + 0.7 (Wang et al. 2024). Once precipitation enters the soil, partial evaporation can occur, causing the residual soil water to become enriched in heavy isotopes through evaporative fractionation (Sprenger et al. 2016). As a result, the *δ* ^2^H–*δ* ^18^O relationship of water that has undergone kinetic fractionation due to evaporation will deviate from both the GMWL and LMWL (Sprenger et al. 2016).

#### 2.2.4 Classification of the Potential Water Sources

Based on observed root distribution patterns and vertical profiles of soil water δ^18^O, we classified potential water sources into three depth‐defined layers: (i) shallow (0–40 cm), characterized by pronounced *δ* ^18^O enrichment and high root biomass; (ii) middle (40–140 cm), exhibiting relatively stable *δ* ^18^O values and a gradual decline in root biomass; and (iii) deep (140–260 cm), where *δ* ^18^O remained stable and no roots were detected. This stratification integrates hydrological properties with biologically accessible soil moisture.

To quantify the proportional contributions of potential water sources to plant xylem water, we applied the Bayesian mixing model MixSIAR (R package v.4.4.1). The model accounts for uncertainty in the isotope values of the mixture (plant xylem water) and source contributions (soil water), including uncertainties arising from over‐parameterization (Moore and Semmens 2008). The fractionation factor was set to zero; parameters for Markov Chain Monte Carlo sampling, priors, and error structures were configured as “long,” “uninformative prior,” and “process only,” respectively. Model convergence was verified prior to generating outputs (Wang et al. 2017).

### 2.3 Data Analysis

Statistical analyses were performed using SPSS 21.0 (IBM Inc., Armonk, NY, USA). Normality of residuals and homogeneity of variances were assessed prior to analysis; log transformations were applied as needed to meet parametric assumptions. Differences in proportional water source use among species were evaluated using one‐way ANOVA, followed by least significant difference (LSD) post hoc tests (*α* = 0.05). Interspecific comparisons of leaf *δ* ^13^C values and proportional water uptake from each soil layer were performed using independent‐samples *t*‐tests.

To analyze root biomass distribution across soil layers, a linear mixed‐effects model (LMM) was constructed with species, soil layer depth, and their interaction included as fixed effects. Individual plant ID was specified as a random intercept to account for repeated measurements across depths within the same individual. To account for the spatial autocorrelation of repeated measurements across soil layers, a first‐order autoregressive (AR(1)) covariance structure was adopted. Significance tests for fixed effects were performed using Type III F‐tests with degrees of freedom approximated by the Satterthwaite method. The significance level was set at *α* = 0.05 for all analyses.

Allometric relationships between root and shoot biomass, as well as ontogenetic changes in root: shoot ratio, were examined using linear regression on log‐transformed data. All data are reported as mean ± standard error (SE). Figures were generated using Origin 2021 (Origin Lab Corp., Northampton, MA, USA).

## 3 Result and Analysis

### 3.1 Soil Water Content and *δ* ^18^O Profiles

**FIGURE 2:**
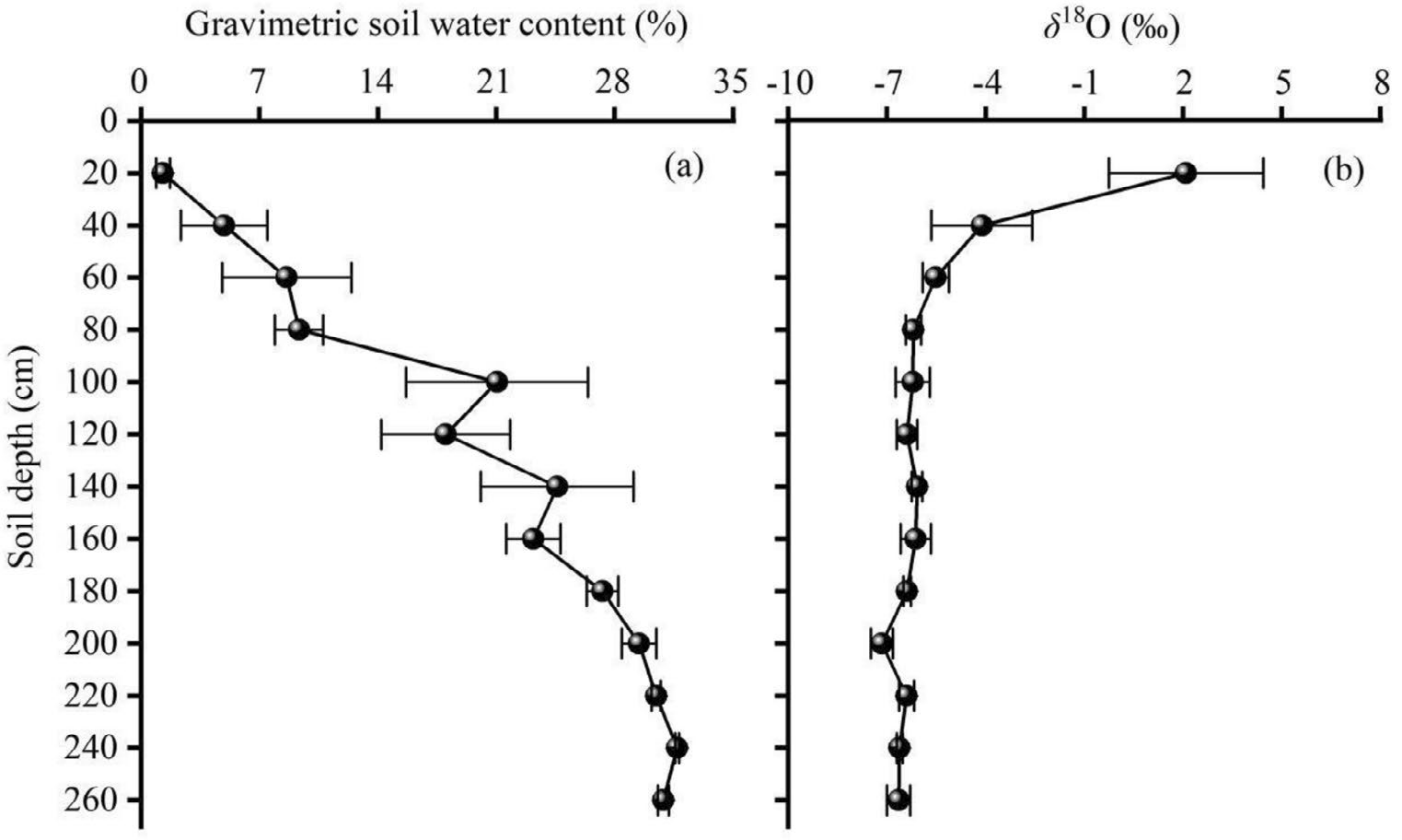
Vertical soil profiles illustrating the gravimetric soil water content (a) and *δ* ^18^O values (b) at the Daliyaboyi Oasis, located in the hinterland of the Taklamakan Desert. Data are mean ± SE; *n* = 3 replicate cores per depth layer.

Soil water content (SWC) increased progressively with depth, rising from 1.29% at 20 cm to 31.68% at the phreatic layer (240 cm; Figure 2a). In contrast, *δ* ^18^O values declined exponentially with depth, exhibiting the highest isotopic enrichment in the 0–40 cm layer and stabilizing below 40 cm (Figure 2b), consistent with strong evaporative fractionation in surface soils.

### 3.2 Isotopic Composition of Water Sources and Xylem Water

**FIGURE 3:**
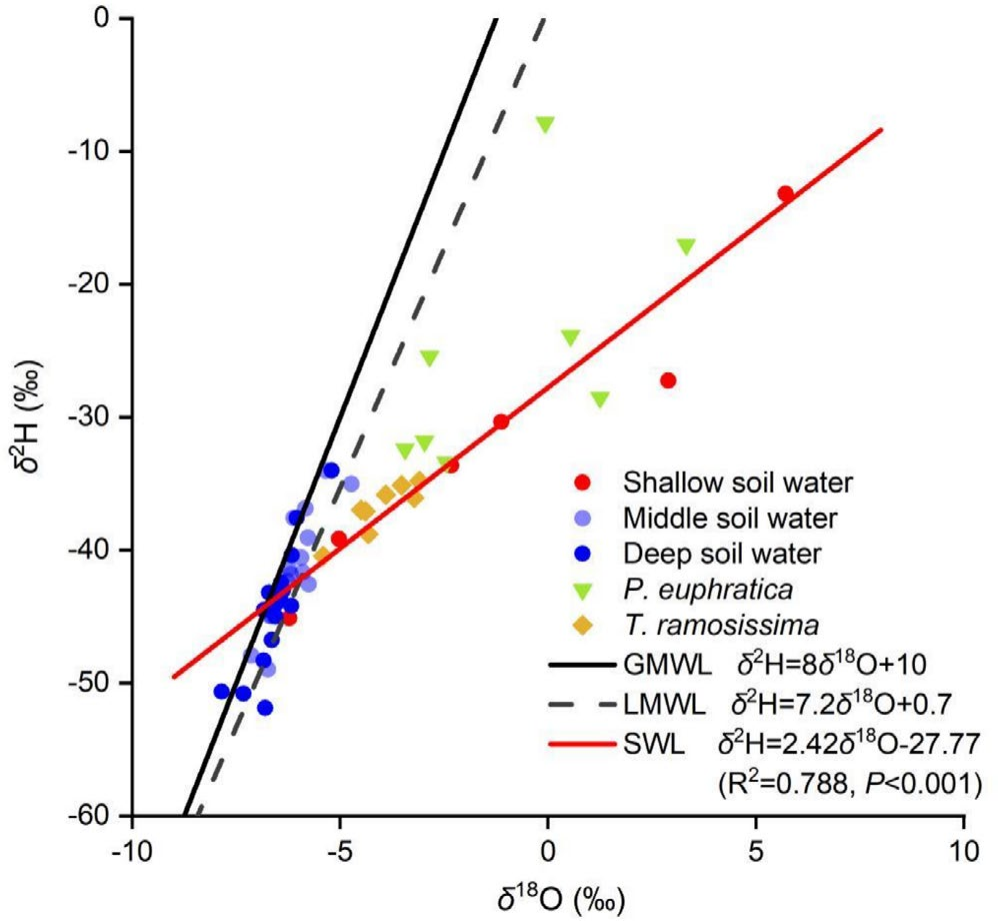
Stable isotope composition of plant xylem water and soil water samples collected in July 2022 at the Daliyaboyi Oasis. The global meteoric water line (GMWL: *δ* ^2^H = 8*δ* ^18^O + 10, solid black line) is derived from Craig (1961); the local meteoric water line (LMWL: *δ* ^2^H = 7.2*δ* ^18^O + 0.7, dashed black line) is sourced from Wang et al. (2024). The soil water line (SWL, solid red line) was fitted based on 39 soil water samples (three replicates across 13 depth intervals). For plant xylem water, *n* = 8 per species.

The *δ* ^2^H and *δ* ^18^O values of soil water ranged from −51.88‰ to −13.19‰ and from −7.85‰ to 5.71‰, respectively (Figure 3). Shallow soil water (0–40 cm) plotted distinctly below both the GMWL and the LMWL, confirming pronounced evaporative enrichment. In contrast, water from the middle (40–140 cm) and deep (140–260 cm) layers clustered closer to the meteoric water lines. The soil water line (SWL) exhibited a slope of 2.42, further corroborating substantial evaporation effects. Xylem water isotopic signatures of both species aligned closely with the soil water line (Figure 3).

### 3.3 Water Source Partitioning

**FIGURE 4:**
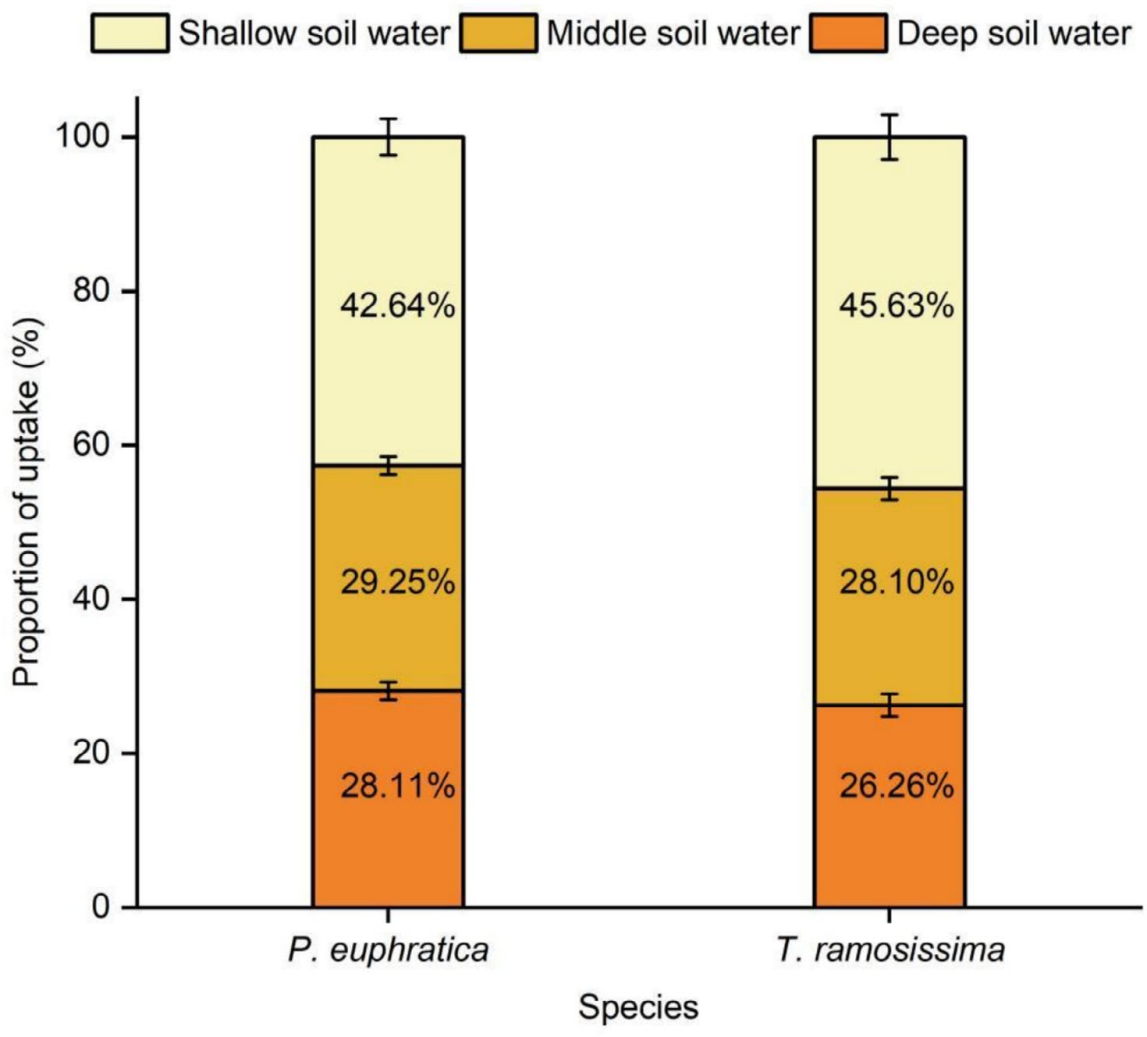
Contribution of potential water sources to *P. euphratica* and *T. ramosissima* seedlings based on MixSIAR analysis. Data are mean ± SE; *n* = 8 per species.

The average water uptake proportions of *P. euphratica* from shallow, middle, and deep soil layers were 42.64% ± 2.37%, 29.25% ± 1.19%, and 28.11% ± 1.18%, respectively, while those of *T. ramosissima* were 45.63% ± 2.92%, 28.10% ± 1.48%, and 26.26% ± 1.44%, respectively (Figure 4). Both species exhibited significantly greater reliance on shallow soil water than on middle or deep soil sources (both *p* < 0.001). No significant interspecific differences were observed in the utilization percentages of shallow (*p* = 0.44), middle (*p* = 0.555), or deep (*p* = 0.338) soil water.

### 3.4 Leaf *δ* ^13^C and Water Use Efficiency

**FIGURE 5:**
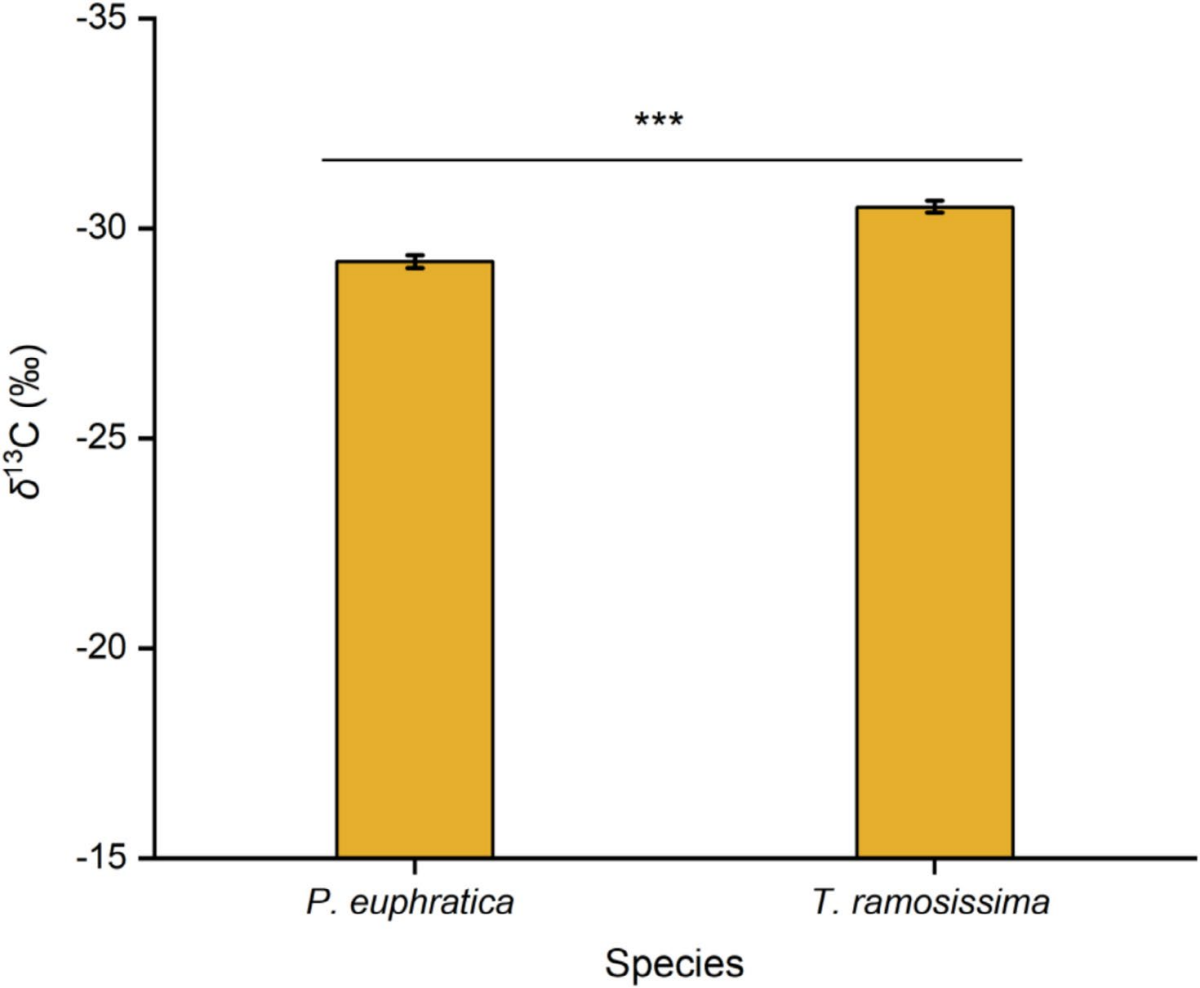
Leaf *δ* ^13^C values of *P. euphratica* and *T. ramosissima* seedlings. Data are mean ± SE; *n* = 6 per species. Asterisks indicate significant difference between species (independent‐sample *t*‐test, ***p < 0.001).

Leaf *δ* ^13^C values were significantly higher in *P. euphratica* (−29.21‰ ± 0.15‰) than in *T. ramosissima* (−30.51‰ ± 0.14‰; *p* < 0.001; Figure 5), indicating that *P. euphratica* seedlings maintain higher WUE under comparable soil moisture conditions.

### 3.5 Root Distribution and Biomass Allocation Patterns

**FIGURE 6:**
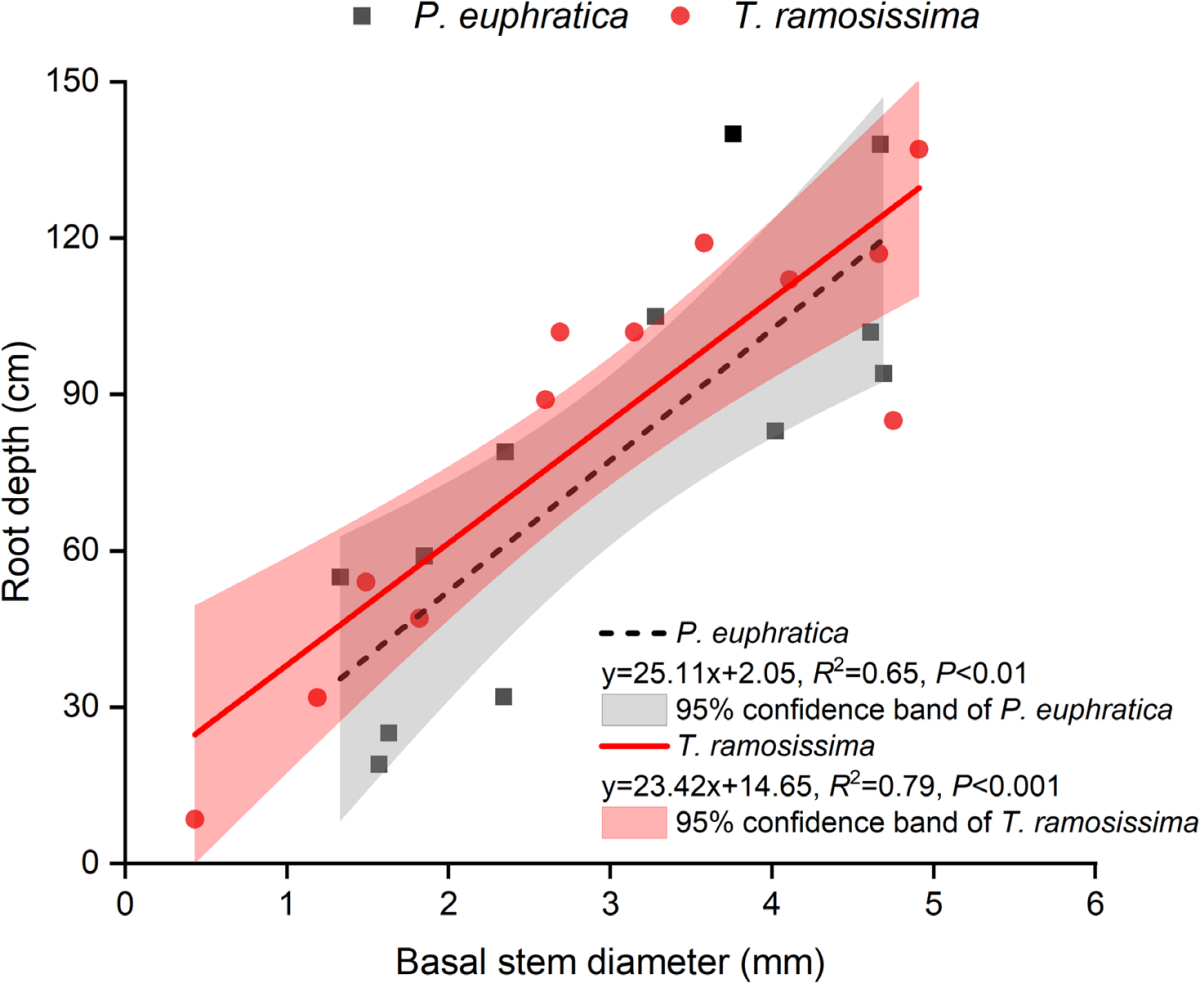
Relationships between basal stem diameter and root depth for *P. euphratica* (black) and *T. ramosissima* (red). Solid and dash lines represent linear regressions; shaded areas indicate 95% confidence bands. For *P. euphratica*: *R* ^2^ = 0.65, *p* < 0.01; for *T. ramosissima* : *R* ^2^ = 0.79, *p* < 0.001. *n* = 12 per species.

Linear regression analysis showed that root depth increased significantly with basal stem diameter for both species (Figure 6; *p* < 0.01 for both). *T. ramosissima* exhibited a stronger linear relationship (*R* ^2^ = 0.79, *p* < 0.001) with a slope of 23.42, indicating a 23.42 cm increase in root depth per 1 mm increase in basal diameter. *P. euphratica* showed a slightly weaker fit (*R* ^2^ = 0.65, *p* < 0.01) but a steeper slope of 25.11. Notably, the intercept for *T. ramosissima* (14.65) was substantially higher than that for *P. euphratica* (2.05), and the 95% confidence intervals did not overlap, indicating consistently deeper rooting in *T. ramosissima* at a given basal diameter.

**FIGURE 7:**
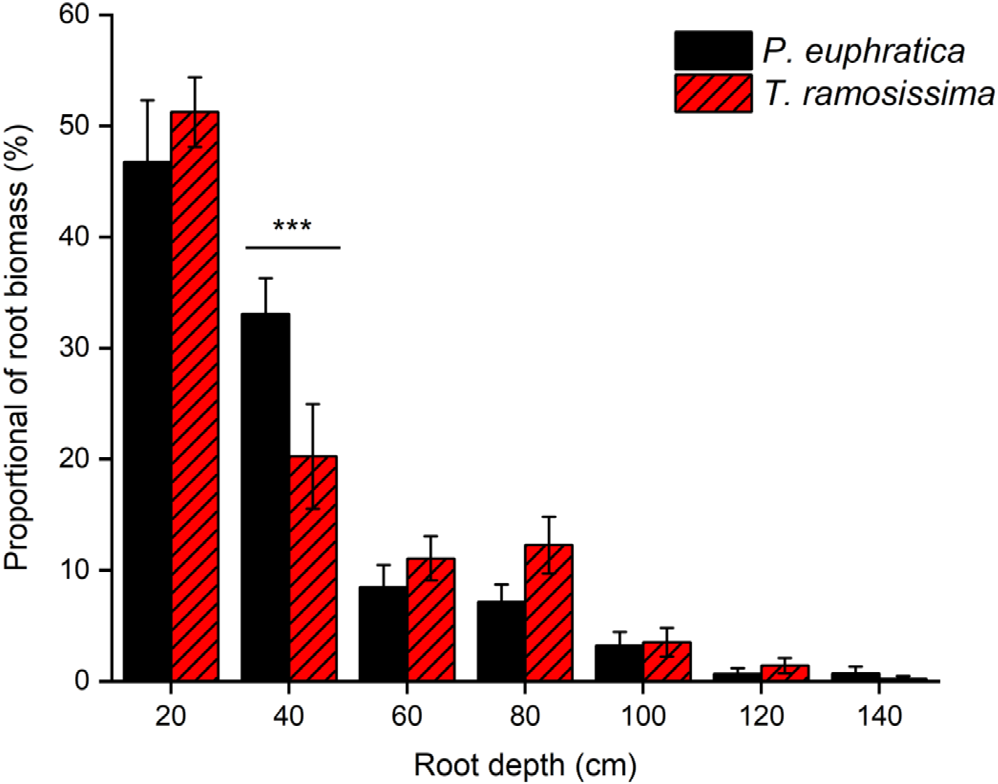
Vertical distribution of root biomass across soil layers for *P. euphratica* and *T. ramosissima* seedlings. Data are mean ± SE; *n* = 8 per species. A significant species × soil layer interaction was detected (linear mixed‐effects models, *F*_6, 59.74_ = 2.61, *p* = 0.026). Asterisks indicate significant differences between species within the same soil layer (*p* < 0.05). Significant differences were observed only in the 20–40 cm layer (***p < 0.001).

Linear mixed‐effects models revealed a highly significant main effect of soil layer on root biomass distribution (*F*_6, 59.74_ = 124.58, *p* < 0.001; Table 2), whereas the main effect of species was not significant (*F*_1, 58.83_ = 0.00, *p* = 1.000). Both species exhibited a typical “surface‐enriched, deep‐depleted” vertical distribution pattern (Figure 7). Root biomass was concentrated in the 0–40 cm layer, accounting for 79.83% of total root biomass in *P. euphratica* and 71.52% in *T. ramosissima* . The species × soil layer interaction was significant (*F*_6, 59.74_ = 2.61, *p* = 0.026). In the 20–40 cm layer, *P. euphratica* had a significantly higher root biomass proportion (33.08%) than *T. ramosissima* (20.26%; *p* < 0.001). No significant differences were detected in other layers, suggesting partial vertical niche differentiation in root distribution between the two species.

**TABLE 2:** Type III *F*‑test results for fixed effects of root biomass distribution based on linear mixed‑effects models.

Source of variation	df_1_	df_2_	*F* value	*p*
Intercept	1	58.83	763.42	< 0.001***
Species	1	58.83	0.00	1.000 ^ns^
Soil layer	6	59.74	124.58	< 0.001***
Species × soil layer	6	59.74	2.61	0.026*

*Note:* df_1_ = numerator degrees of freedom; df_2_ = denominator degrees of freedom (approximated by Satterthwaite method); significance codes: *** *p* < 0.001, ** *p* < 0.01, * *p* < 0.05, ns = not significant (*p* ≥ 0.05).

**FIGURE 8:**
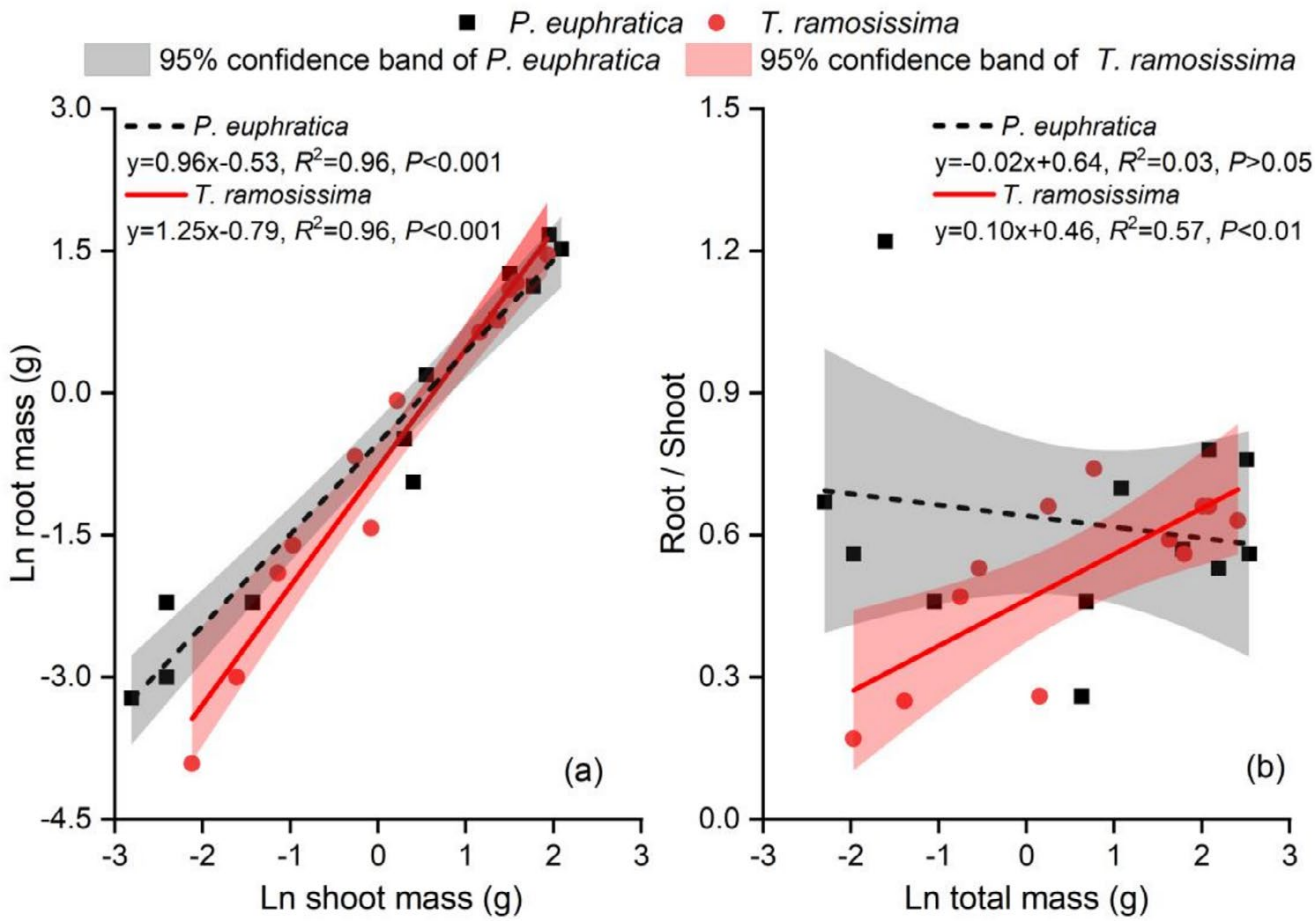
Allometric relationships and biomass allocation patterns of *P. euphratica* and *T. ramosissima* seedlings. (a) Linear regression between ln‐transformed root biomass and shoot biomass; (b) Relationship between root: shoot ratio and ln‐transformed total biomass. Black dashed lines represent *P. euphratica*; red solid lines represent *T. ramosissima* . Shaded areas indicate 95% confidence intervals. *n* = 12 per species.

In log–log space, both species showed strong positive relationships between root and shoot biomass (Figure 8a; *p* < 0.001 for both). The regression slope for *T. ramosissima* (1.25, *R* ^2^ = 0.96) was slightly steeper than that for *P. euphratica* (0.96, *R* ^2^ = 0.96), indicating that root biomass increased faster with shoot biomass in *T. ramosissima* , reflecting a greater relative investment in belowground structures as plants grow. Analysis of root: shoot ratio against total biomass (Figure 8b) revealed contrasting ontogenetic strategies. For *P. euphratica*, the root: shoot ratio showed no significant trend with increasing total biomass (*R* ^2^ = 0.03, *p* > 0.05). In contrast, *T. ramosissima* exhibited a statistically significant positive relationship (*R* ^2^ = 0.57, *p* < 0.01), indicating an ontogenetic shift toward increased belowground biomass allocation with plant growth.

## 4 Discussion

### 4.1 Convergent Water Sources but Contrasting Water Use Efficiency

Consistent with our first hypothesis, *P. euphratica* and *T. ramosissima* seedlings did not exhibit niche partitioning of soil water sources. Instead, both species relied predominantly on shallow soil water (0–40 cm), and no significant interspecific differences in proportional uptake from any soil layer (Figure 4). This convergence in water‐source utilization aligns with the high niche overlap reported for these species during the seedling stage (Li et al. 2013) and reflects the relatively high moisture availability in the upper soil profile (Figure 2a), sustained by seasonal flooding and shallow groundwater in the Daliyaboyi Oasis (Abdureyim et al. 2024; Zhang et al. 2024).

Despite sharing similar water sources, the two species exhibited distinct water‐use efficiency. The significantly higher leaf *δ* ^13^C value in *P. euphratica* (Figure 5) indicates greater long‐term WUE, a trait mechanistically linked to tighter stomatal regulation and reduced carbon assimilation under mild water stress (Ehleringer 1993; Cao et al. 2020). Recent genomic studies have identified drought‐tolerance genes in *P. euphratica* that modulate stomatal development and density, providing a molecular basis for its enhanced WUE (Ndayambaza et al. 2023; Jia et al. 2024). In contrast, *T. ramosissima* maintains lower *δ* ^13^C values, reflecting a more wateracquisitive strategy facilitated by deeproot proliferation (Wang et al. 2021). Our results demonstrated that species‐specific WUE differences between *P. euphratica* and *T. ramosissima* are already evident at the seedling stage under natural coexistence.

### 4.2 Divergent Biomass Allocation Patterns

Our second hypothesis, that the two species diverge in biomass allocation as they grow to reduce competition, was robustly supported by interspecific differences in root distribution and allometric scaling. Linear regression of root depth against basal stem diameter showed that *T. ramosissima* achieved consistently deeper rooting at a given diameter than *P. euphratica*, as indicated by non‐overlapping confidence intervals of the intercepts and a steeper slope for *P. euphratica* (Figure 6). This suggests that while both species increase root depth with size, *T. ramosissima* starts from a deeper baseline.

Mixedeffects models revealed a significant species × soil layer interaction for root biomass distribution. Although the main effect of species was not significant, *P. euphratica* concentrated a larger proportion of its root biomass in the uppermost soil layers, whereas *T. ramosissima* invested more biomass in deeper layers. This preferential deep‐root investment enhances access to stable subsoil water, reducing dependence on evaporatively depleted surface moisture and sustaining leaf water potential under drought (Gries et al. 2003; Li et al. 2013). The rapid vertical elongation of *T. ramosissima* roots confers a competitive advantage in arid environments (Imada et al. 2013; Abdureyim et al. 2024).

Allometric analysis further revealed contrasting ontogenetic strategies. Both species showed strong positive relationships between root and shoot biomass (Figure 8a). The slope for *T. ramosissima* was steeper than that for *P. euphratica*, indicating that root biomass increased faster with shoot biomass in *T. ramosissima* . Analysis of the root: shoot ratio in relation to total biomass (Figure 8b) corroborated this pattern: *P. euphratica* exhibited no statistically significant trend, whereas *T. ramosissima* displayed a significant positive correlation, indicating an increasing allocation of biomass to belowground structures with plant growth. These divergent patterns likely reflect different selective pressures: *P. euphratica* emphasizes light capture and canopy expansion in floodplain environments, whereas *T. ramosissima* adopts a water‐securing strategy through enhanced root investment under variable moisture regimes (Li et al. 2013; Wang et al. 2015).

Our field‐based findings contrast with previous pot experiments, where *T. ramosissima* seedlings allocated proportionally less biomass to roots than *P. euphratica* under favorable groundwater conditions (Wu et al. 2018). This discrepancy likely reflects fundamental differences in growing environment. Pot experiments often restrict root expansion space and homogenize resource distribution, which may suppress the expression of competitive root responses that would otherwise occur under natural conditions (Casper et al. 1998). In heterogeneous field environments, interspecific competition for belowground resources becomes more intense, and plants often respond by enhancing root investment to secure limiting soil water (Kiær et al. 2013). Consistent with this, the present study found that in mixed stands, *T. ramosissima* increased its proportional root biomass allocation to deeper soil layers, indicating that competitive pressure for soil water likely drives this response. More broadly, interspecific interactions in multispecies communities can induce shifts in root depth distribution and biomass allocation, potentially promoting vertical niche differentiation among coexisting species (Wardle and Peltzer 2003; de Kroon et al. 2012).

### 4.3 Implications for Coexistence and Riparian Management

The convergent use of shallow soil water (Hypothesis i) and the divergent biomass allocation patterns (Hypothesis ii) together suggest that *P. euphratica* and *T. ramosissima* seedlings coexist through complementary resource‐use strategies rather than strict niche differentiation. This functional differentiation likely mitigates direct competition, thereby promoting stable coexistence in arid riparian ecosystems (Chesson 2000; Silvertown 2004; Kraft et al. 2015). Recent riparian studies have further shown that hydrological alterations strongly influence competitive dynamics, with species exhibiting complementary strategies tending to maintain higher coexistence stability under fluctuating conditions (Liu et al. 2024).

From a management perspective, these contrasting strategies carry important implications for riparian restoration under changing groundwater regimes. *P. euphratica* seedlings, with their shallower root systems, are more vulnerable to surface soil drying; maintaining shallow soil moisture, via controlled flooding or watertable management, is therefore critical for their successful establishment. In‐ contrast, *T. ramosissima* exhibits greater root plasticity and tolerance to variable water availability, making it a more resilient candidate for restoration in hydrologically uncertain environments (Stromberg et al. 2007; Merritt and Poff 2010). However, the competitive root advantage of *T. ramosissima* could potentially suppress *P. euphratica* regeneration in mixed plantings if not carefully managed. Future restoration initiatives should therefore integrate species‐specific water‐use traits into site selection, allocating planting locations according to local hydrological stability to support the long‐term coexistence of these dominant species.

## 5 Conclusions

This study investigated water source utilization and biomass allocation in cooccurring *P. euphratica* and *T. ramosissima* seedlings in a desert riparian ecosystem of the Taklamakan Desert. Both species relied predominantly on shallow soil water (0–40 cm), supporting our first hypothesis of convergent water source use. However, they exhibited contrasting water‐use efficiency, with *P. euphratica* showing higher leaf *δ* ^13^C values (−29.21‰) than *T. ramosissima* (−30.51‰), indicating greater water‐use efficiency.

In line with our second hypothesis, the two species diverged in biomass allocation during growth. *P. euphratica* increasingly allocated biomass to aboveground tissues, whereas *T. ramosissima* shifted investment toward belowground structures, particularly in deeper soil layers. These complementary strategies, convergent water sourcing but divergent biomass partitioning, likely reduce direct competition and facilitate the stable coexistence of these dominant species in arid riparian zones.

From a management perspective, maintaining shallow soil moisture through natural flood pulses or regulated water releases is critical for *P. euphratica* regeneration, while *T. ramosissima* may serve as a more resilient species under fluctuating hydrological regimes due to its deeper rooting and greater belowground plasticity. Future research should extend beyond this single‐year study to incorporate multi‐year stable isotope monitoring and process‐based modeling, thereby improving predictive capacity for plant community dynamics under projected increases in drought severity and declines in groundwater levels.

## Author Contributions

**Yue Dai:** conceptualization (lead), formal analysis (lead), funding acquisition (lead), writing – original draft (lead). **Zhuanxiong Ye:** data curation (equal), investigation (lead), writing – original draft (equal). **Anwar Abdureyim:** investigation (lead), methodology (equal). **Mengmeng Tang:** writing – review and editing (equal). **Kerui Yao:** writing – review and editing (equal).

## Funding

This work was supported by The “Tianshan Talents” Program of the Science and Technology Department of Xinjiang Uygur Autonomous Region (2024TSYCLJ0006), and the National Natural Science Foundation of China (32160260).

## Conflicts of Interest

The authors declare no conflicts of interest.

## Data Availability

All data that support the findings of this study are deposited in the Dryad Digital Repository: https://doi.org/10.5061/dryad.ngf1vhj8v. No additional Supporting Information files are included with this article.
